# Optimizing health workforce distribution and satisfaction in Ghana’s Upper East Region: a quantitative analysis of intrinsic and extrinsic predictors

**DOI:** 10.11604/pamj.2026.54.13.48260

**Published:** 2026-05-18

**Authors:** Augustina Memang, Richmond Balinia Adda, Simon Subinleeb Bawa, Kofi Akohene Mensah

**Affiliations:** 1 Department of Health Policy, Management and Economics, School of Public Health, Kwame Nkrumah University of Science and Technology, Kumasi, Ghana,; 2 Navrongo Health Research Centre, Ghana Health Service, Navrongo, Ghana

**Keywords:** Health workforce distribution, job satisfaction, primary healthcare, Ghana, rural retention, supervision, health policy

## Abstract

**Introduction:**

inadequate health workforce distribution and retention challenges hinder Universal Health Coverage in low-income settings. This study assessed health worker distribution and satisfaction determinants in public primary healthcare facilities in Ghana's Upper East Region.

**Methods:**

a quantitative cross-sectional design was employed. Data were collected from human resource departments and health workers via semi-structured questionnaires. Univariate and multivariate regression analyses identified associations between workforce satisfaction and intrinsic/extrinsic factors.

**Results:**

significant staffing gaps were observed: Bawku West (27.99%), Builsa North (36.94%), and Bongo (51.67%). Multivariate analysis revealed that clear responsibility (aOR=4.58, p=0.001) and effective communication (aOR=10.14, p<0.001) were strong intrinsic predictors of satisfaction. Enhanced supervision (aOR=8.39, p<0.001) was a key extrinsic factor. Female workers had lower satisfaction odds (aOR=0.70), while widowed/separated individuals reported higher satisfaction (aOR=2.15).

**Conclusion:**

targeted interventions to clarify roles, improve communication, and strengthen supervision are critical to optimize workforce distribution and enhance satisfaction in rural Ghana.

## Introduction

Achieving Universal Health Coverage (UHC) remains a pressing challenge for many low- and middle-income countries, primarily due to inadequate and inefficient health workforce distribution [1,2]. In Ghana, disparities in health worker deployment—particularly between urban centers and rural districts—continue to undermine the delivery of equitable and quality healthcare services [3-5]. Despite national strategies such as the staffing norm framework and the Workload Indicators of Staffing Needs (WISN) methodology [6], regions like the Upper East still face chronic shortages of health professionals [7,8]. These shortages are further compounded by retention issues and low job satisfaction among healthcare workers, which are influenced by both intrinsic factors (e.g., responsibility, recognition, communication) and extrinsic conditions (e.g., supervision quality, work environment, interpersonal relationships) [9-11]. The Upper East Region—characterized by its rural profile, limited infrastructure, and disproportionate disease burden—offers a critical setting to examine these challenges in detail [7]. Earlier studies have demonstrated not only a substantial quantitative gap in staffing, but also qualitative deficiencies linked to motivation and job satisfaction in this region [3,12]. Addressing these issues requires understanding the determinants that encourage health workers to remain in their roles and the mechanisms through which job satisfaction affects retention and service delivery [13,14]. This study draws on theoretical frameworks such as the Standard Allocation Theory and Herzberg's Two-Factor Theory [15] and applies a quantitative cross-sectional design to examine the distribution of the health workforce and predictors of job satisfaction in public primary healthcare facilities in Ghana's Upper East Region. Using integrated data from human resource departments and health worker surveys, the research explores both intrinsic and extrinsic motivators to inform targeted policy interventions aimed at improving workforce distribution, enhancing satisfaction, and strengthening healthcare delivery in underserved settings.

## Methods

### Study design and population

A quantitative cross-sectional design was employed to assess both the distribution of the health workforce and the determinants of job satisfaction in public primary healthcare facilities in the Upper East Region of Ghana. The study population comprised clinical health workers, including nurses, doctors, pharmacists, laboratory technicians, and other frontline clinical staff, as well as human resource managers operating within public health facilities. Participants were selected using multistage sampling techniques, which involved clustering health facilities by geographical zones (Eastern, Central, and Western) within the region and then employing simple random sampling to select individual facilities and participants. Of 423 eligible health workers, 385 participated (91.0% response rate). Primary reasons for non-participation included being off-duty during data collection (n=22, 5.2%), declined due to workload constraints (n=12, 2.8%) and administrative transfer during the study period (n=4, 0.9%). The study size was determined by a multistage sampling approach targeting ≥ 80% of clinical staff across all public primary facilities in three purposefully selected high-shortage districts (Bongo, Bawku West, Builsa North). This achieved a 95% confidence level with a 5% margin of error based on regional workforce data [16].

### Study setting and data collection

The study was conducted across selected districts in the Upper East Region, a predominantly rural area marked by significant health service challenges and workforce deficits. Data were collected from two primary sources: secondary data from human resource departments, which provided information on staffing levels, gaps in personnel relative to Ministry of Health staffing norms, and facility-specific workload indicators, and primary data through self-administered semi-structured questionnaires distributed directly to health workers. We used the structured questionnaire for data collection. These questionnaires gathered detailed information on intrinsic factors (such as recognition, responsibility, career advancement, work schedule, and communication) and extrinsic factors (including supervision quality, work environment, salary satisfaction, interpersonal relationships, and organizational support) that may influence workforce satisfaction. Additional socio-demographic data (e.g., age, sex, marital status, education, work experience) were also collected to allow for an in-depth analysis of the predictors of job satisfaction.

**Study variables:** the primary outcome variable for the study was health workforce satisfaction. This was measured using a set of indicators capturing both intrinsic factors (personal achievement, recognition, responsibility, career advancement, work schedule, and communication quality) and extrinsic factors (work environment, supervision, salary satisfaction, interpersonal relationships, and organizational support). Other study variables included socio-demographic characteristics such as age, gender, marital status, educational level, occupation, and years of work experience. Staffing gap data is defined as the discrepancy between actual staffing and the Ministry of Health´s recommended staffing norms, which were also analysed as an indicator of workforce distribution inefficiencies.

**Statistical analysis:** data were entered and analysed using SPSS (version 26). Descriptive statistics (frequencies, percentages, means, and standard deviations) were used to summarize the demographic characteristics of the study population and the distribution of staffing gaps. Univariate regression analysis was performed to identify associations between individual intrinsic and extrinsic factors and workforce satisfaction. To control for potential confounders and assess the independent effects of each predictor, multivariate regression analyses (ordered logistic regression models) were conducted. Prespecified subgroup analyses by occupation (nurses vs. non-nurses), district, and work experience (= 3 vs. >3 years) were conducted using interaction terms in regression models. Effect modification was tested via likelihood ratio tests comparing models with/without interaction terms. Missing questionnaire responses (2.1% of total data points) were excluded pairwise from regression analyses. Sensitivity analysis confirmed exclusion did not alter effect estimates by >5%. Statistical significance was determined at the 0.05 level.

### Data availability statement

The datasets generated and/or analyzed during the current study are not publicly available due to ethical restrictions related to participant confidentiality. However, anonymized data may be made available upon reasonable request from the corresponding author, subject to approval by the Committee for Human Research Publications and Ethics (CHRPE) at the Kwame Nkrumah University of Science and Technology (KNUST), Ghana.

**Ethics approval:** the study protocol was reviewed and approved by the Committee for Human Research Publication and Ethics (CHRPE) with the clearance number CHRPE/AP/507/22. Informed consent was obtained from all participants after they were briefed about the study´s objectives, procedures, potential risks, and benefits. Participants were assured that their responses would remain confidential and that their participation was entirely voluntary, with the freedom to withdraw at any point without any negative consequences.

## Results

### Demographic characteristics and workforce distribution

Table 1 provides a detailed breakdown of the demographic profile of the study respondents, offering valuable insights into the composition of the health workforce in the Upper East Region. Missing data per variable: responsibility (0.8%), communication (1.2%), supervision (0.5%), salary (1.6%). Complete case percentages exceeded 98% for all regression predictors. A key observation from the table is the predominance of female respondents, who represent 58.70% of the sample compared to 41.30% of males. This gender distribution suggests that women form most of the frontline health workforce in this region. The age distribution indicates a youthful workforce, with 78.40% of respondents aged between 20 and 30 years. This could imply both a high level of adaptability and potential challenges related to limited professional experience, as only 17.70% fall within the 31-40 years bracket and a mere 3.90% are between 41 and 50 years. Marital status data reveal that 63.90% of respondents are married, while 34.50% are single. The small percentages of separated (0.30%) and widowed (1.30%) individuals reflect a stable family structure among most respondents, which might influence retention and job satisfaction dynamics. Regarding educational attainment, over half of the respondents (51.95%) hold certificate-level qualifications. Diploma holders account for 28.57%, while those with a first degree and master´s degree comprise 11.69% and 7.79%, respectively. This distribution highlights that the workforce is largely composed of individuals with lower-level qualifications, which could have implications for service quality and opportunities for career advancement. In terms of occupational roles, nurses form the overwhelming majority at 79.20%, with doctors, laboratory technicians, and HR managers representing much smaller segments (1.00%, 1.30%, and 3.60%, respectively). This indicates a heavy reliance on nursing professionals in primary healthcare settings. The religious composition is predominantly Christian (74.80%), followed by Muslim (21.30%) and Traditional (3.90%) affiliations, reflecting the broader religious demographics of the region. Monthly income levels reveal that 44.16% of respondents earn between 1001 and 1500 GHC, with the smallest group (5.20%) earning above 3000 GHC. This income distribution suggests that most of the workforce operates within modest salary ranges, which may affect job satisfaction and retention. Finally, work experience is generally limited, with 58.20% of respondents reporting three years or less. This reinforces the finding of a youthful, relatively inexperienced workforce, potentially necessitating targeted strategies for professional development and mentorship.

**Table 1:** demographic characteristics of respondents

Characteristics	Frequency	Percentage (%)
**Sex**		
Male	159	41.3
Female	226	58.7
Age		
20--30 years	302	78.4
31--40 years	68	17.7
41--50 years	15	3.9
**Marital status**		
Married	246	63.9
Single	133	34.5
Separated	1	0.3
Widowed	5	1.3
**Educational level**		
Certificate	200	51.95
Diploma	110	28.57
First Degree	45	11.69
Master's Degree	30	7.79
**Occupation**		
Doctor	4	1.0
Nurse	305	79.2
Laboratory Technician	5	1.3
HR Manager	14	3.6
Others	57	14.8
Religion		
Christian	288	74.8
Muslim	82	21.3
Traditional	15	3.9
**Monthly income (GHC)**		
1001--1500	170	44.16
1501--2000	100	25.97
2001--2500	50	12.98
2501--3000	45	11.69
> 3000	20	5.2
**Work experience**		
≤ 3 years	224	58.2
4--7 years	72	18.7
8--11 years	51	13.2
≥ 11 years	38	9.9

**Source:** Field data (2024)

### Staffing gaps

Staffing gaps across three districts are presented in Table 2. In Bongo District, the staffing gap is 51.67%. This high percentage indicates a severe shortage, meaning that over half of the required health workforce is missing. Such a significant deficit could severely compromise service delivery, leading to increased workloads for the existing staff and potentially lower quality of care for patients. Bawku West District shows a staffing gap of 27.99%. Although lower than Bongo, this moderate gap still highlights a substantial shortfall in the number of health professionals relative to the established norms. This gap, while less dramatic, still poses challenges in maintaining optimal healthcare services and may result in compromised patient care and overburdened staff. Builsa North District has a staffing gap of 36.94%, placing it between Bongo and Bawku West in terms of workforce deficiency. This level of gap suggests that while some facilities might manage with the available staff, there remains a critical need for additional health workers to meet the service demands and ensure effective healthcare delivery.

**Table 2:** staffing gaps relative to ministry of health norms

District	Staffing Gap (%)
Bongo	51.67
Bawku West	27.99
Builsa North	36.94
Source: Field data (2024)	

* Note: Staffing gap = [(Recommended Staff - Actual Staff) / Recommended Staff] x 100*

### Intrinsic factors influencing workforce satisfaction

The intrinsic factors analysis (Table 3 and Table 4) shows that while several factors were significant in univariate and multivariate regression, only a few maintained significance in the multivariate model. Notably, Responsibility and Communication emerged as robust, independent predictors of workforce satisfaction. In the multivariate analysis, responsibility had an odds ratio of 4.58 (p = 0.001; 95% CI: 1.81-11.60), indicating that health workers who feel clear accountability and ownership in their roles are over four times more likely to be satisfied. Similarly, effective communication was highly influential, with an odds ratio of 10.14 (p < 0.001; 95% CI: 3.77-27.29), suggesting that clear and open communication channels significantly boost job satisfaction. Although Recognition, Career Advancement, and Work Schedule/Workload were significant in the univariate model, their effects diminished when controlling for other factors, underscoring the dominant role of responsibility and communication in influencing satisfaction.

**Table 3:** univariate regression analysis of intrinsic factors influencing workforce satisfaction

Factor	Odds Ratio	P-value	95% CI
Achievement	1.43	0.222	0.80-2.54
Recognition	2.17	0.010	1.20-3.90
Responsibility	3.22	<0.001	1.69-6.11
Career Advancement	1.84	0.038	1.03-3.26
Work Schedule	5.02	0.002	1.80-14.00
Communication	13.31	<0.001	5.60-31.61

**Table 4:** multivariate regression analysis of intrinsic factors influencing workforce satisfaction

Factor	Odds Ratio	P-value	95% CI
Achievement	0.58	0.166	0.27-1.25
Recognition	1.13	0.776	0.50-2.59
Responsibility	4.58	0.001**	1.81-11.60
Career Advancement	0.74	0.429	0.35-1.57
Work Schedule	2.05	0.258	0.59-7.11
Communication	10.14	<0.001***	3.77-27.29

Source: Field data (2024) **p<0.05, **p<0.01, ***p<0.001*

### Extrinsic factors influencing workforce satisfaction

Table 5 and Table 6 present the extrinsic factors affecting workforce satisfaction. Here, Supervision stands out as the most significant factor. In the multivariate model, the odds ratio for supervision was 8.39 (p < 0.001; 95% CI: 3.07-22.92), reflecting that adequate supervision greatly enhances job satisfaction by providing necessary support and guidance. Organization and administration also maintained a strong significant association, with an odds ratio of 3.29 (p < 0.001; 95% CI: 1.68-6.43), indicating that effective administrative practices contribute substantially to a supportive work environment. Although Working Environment and Salary showed significance in the univariate analysis, their impact was not statistically significant in the adjusted multivariate model. Similarly, while interpersonal relationships had a strong univariate association (OR = 4.19, p = 0.001), its effect was attenuated in the multivariate analysis (OR = 2.09, p = 0.126), suggesting that its influence may overlap with or be mediated by other factors such as supervision.

**Table 5:** univariate regression analysis of extrinsic factors influencing workforce satisfaction

Factor	Odds Ratio	P-value	95% CI
Working Environment	2.85	0.002**	1.45-5.61
Supervision	11.28	<0.001***	4.99-25.54
Salary	1.53	0.415	0.55-4.25
Interpersonal Relationships	4.19	0.001**	1.78-9.85
Organization/Administration	3.29	<0.001***	1.68-6.43

Source: Field data (2024) ** p<0.05, ** p<0.01, *** p<0.001*

**Table 6:** multivariate regression analysis of extrinsic factors influencing workforce satisfaction

Factor	Odds Ratio	P-value	95% CI
Working Environment	1.26	0.550	0.57-2.70
Supervision	8.39	<0.001***	3.07-22.92
Salary	1.56	0.383	0.58-4.21
Interpersonal Relationships	2.09	0.126	0.81-5.36
Organization/Administration	3.29	<0.001***	1.68-6.43

Source: Field data (2024) ** p<0.05, ** p<0.01, *** p<0.001*

### Sensitivity analyses

Sensitivity analyses confirmed the robustness of primary findings. Exclusion of facilities with staffing gaps exceeding 40% yielded virtually unchanged estimates for responsibility (aOR = 4.61, 95% CI [1.79, 11.85]) and supervision (aOR = 8.44, 95% CI [3.09, 23.04]). Multiple imputation for missing data produced minimal changes in effect sizes (ΔOR ≤ 0.15 for all significant predictors). Prespecified subgroup analyses indicated no significant effect modification by occupation or district (interaction ps > .20).

### Supplementary analyses

Subgroup analysis by occupation (nurses vs. other clinical staff) showed consistent effects of responsibility (aOR=4.21-4.93) and communication (aOR=9.87-10.31). Sensitivity analyses excluding facilities with >40% staffing gaps yielded unchanged multivariate estimates (aOR<0.2).

## Discussion

This study provides crucial insights into determinants of workforce satisfaction among health workers in public primary healthcare facilities in Ghana's Upper East Region. Our analysis reveals the complex nature of job satisfaction, showing that both intrinsic and extrinsic factors contribute significantly to workforce morale, though with varying levels of influence [17,18]. The investigation of intrinsic factors —including achievement, recognition, responsibility, career advancement, work schedule, and communication— yielded important findings. While univariate analysis identified several significant associations, multivariate adjustment demonstrated that only responsibility (aOR = 4.58, p = 0.001; 95% CI: 1.81-11.60) and communication (aOR = 10.14, p < 0.001; 95% CI: 3.77-27.29) maintained independent predictive value for workforce satisfaction. The strong association with responsibility supports Herzberg's Two-Factor Theory [19], confirming that intrinsic job elements such as role clarity serve as powerful motivators in healthcare settings [20,21]. The particularly robust effect of communication underscores its fundamental importance in healthcare environments, where clear information exchange is essential for both patient safety and staff morale [22,23]. This finding emphasizes that transparent communication facilitates effective teamwork while ensuring staff understand their roles and responsibilities, thereby reducing ambiguity and enhancing job satisfaction [24].

Analysis of extrinsic factors revealed equally compelling results. Supervision emerged as the most significant extrinsic predictor (aOR = 8.39, p < 0.001; 95% CI: 3.07-22.92), highlighting the critical importance of supportive leadership in fostering positive work environments [25,26]. This finding is particularly relevant in resource-constrained settings like the Upper East Region, where effective supervision may help mitigate challenges posed by staffing shortages and heavy workloads [27,28]. Additionally, organizational and administrative practices maintained significant association with satisfaction (aOR = 3.29, p < 0.001; 95% CI: 1.68-6.43), suggesting that streamlined administrative processes contribute substantially to a supportive work environment [29,30]. Notably, while working environment, salary, and interpersonal relationships showed significance in univariate analysis, their effects attenuated in multivariate models. This suggests that although these factors remain important considerations for health workforce management [31,32], their independent effects may be mediated through more proximal factors such as supervision quality and organizational efficiency [33,34].

These findings have substantial implications for policy and practice in rural healthcare settings. They advocate for a dual approach addressing both intrinsic and extrinsic factors simultaneously. For intrinsic factors, interventions should focus on clarifying role definitions and enhancing communication channels through regular feedback mechanisms and team-building activities [35,36]. For extrinsic factors, investments in leadership development, supervisory training, and administrative reforms appear crucial [17]. This comprehensive approach may prove particularly valuable in addressing the chronic workforce challenges observed in rural Ghana [4,15] and similar settings across low- and middle-income countries [1,11]. The strong effects of communication and supervision identified in this study suggest that relatively low-cost interventions targeting these areas could yield substantial improvements in workforce satisfaction and retention [14,32]. Future interventions should consider implementing structured communication protocols, regular supervisory training, and clear accountability frameworks as potential strategies for improving workforce satisfaction in resource-constrained settings.

**Study limitations:** this study´s limitations include its cross-sectional design, which prevents establishing causality between variables. The research was conducted exclusively in public primary healthcare facilities in the Upper East Region of Ghana, limiting the generalizability of the findings to other regions and healthcare settings, including private or specialized facilities. Data collection relied on self-administered questionnaires, which may be subject to response biases such as social desirability. Additionally, while several intrinsic and extrinsic factors were examined, the study did not capture all potential determinants of workforce satisfaction.

## Conclusion

This study provides critical insights into the challenges of health workforce distribution and job satisfaction within public primary healthcare facilities in the Upper East Region of Ghana. Our findings reveal significant staffing gaps, particularly in rural districts, which threaten the timely delivery of quality healthcare services. Through robust univariate and multivariate analyses, we identified key intrinsic factors, specifically responsibility and effective communication, that independently drive workforce satisfaction. Similarly, among extrinsic factors, supportive supervision and efficient organization and administration emerged as essential contributors to a positive work environment. These results underscore the need for targeted policy interventions that not only address the quantitative deficits in staffing but also enhance the qualitative aspects of workforce management. Strengthening supervisory practices, clarifying role responsibilities, and fostering open communication can collectively improve job satisfaction, thereby promoting retention and optimal service delivery. Future research should adopt a longitudinal approach to monitor the impact of such interventions, ensuring that the evolving needs of the health workforce are met sustainably. We recommend Ghanaian policymakers: 1) Implement mandatory role clarification frameworks across primary facilities to define responsibilities; 2) Establish structured communication channels (e.g., monthly feedback sessions between staff/supervisors); 3) Integrate supportive supervision training into Ghana Health Service retention programs. Strengthening these areas can collectively improve job satisfaction, promote retention, and optimize service delivery.

### What is known about this topic

- Health worker shortages remain a major bottleneck to Universal Health Coverage in Ghana;
- Prior studies have highlighted the role of job satisfaction in workforce retention;
- Limited empirical evidence exists on rural Ghana´s intrinsic and extrinsic satisfaction factors.

### What this study adds

- This study quantifies staffing gaps in rural Ghana using updated regional data;
- It identifies communication, supervision, and responsibility as key satisfaction drivers;
- Results will guide tailored interventions to retain frontline health workers.
